# Vocal Hoarseness and a Subglottic Mass: An Uncommon Diagnosis for a Common Complaint

**DOI:** 10.1177/2324709615587528

**Published:** 2015-05-19

**Authors:** Sassan Rafizadeh, Ken Yoneda, Amir A. Zeki

**Affiliations:** 1 University of California, Davis Medical Center, Sacramento, CA, USA

**Keywords:** tracheopathia osteoplastica, hoarseness, airway stenosis, trachea, osteocartilaginous nodules

## Abstract

We report a patient with tracheopathia osteoplastica (TPO), a rare or perhaps underrecognized disorder, detected in approximately 1 in every 2000 to 5000 patients who undergo bronchoscopy. TPO is marked by proliferation of bony and cartilaginous spurs leading to airway stenosis. Multiple submucosal cartilaginous and osseous nodules can develop in the respiratory tract and may involve the entire trachea and mainstem bronchi. Symptoms may range from a completely silent condition to life-threatening respiratory failure and diagnosis is made based on radiological and bronchoscopic findings. Although the etiology has not been established, TPO can be familial and is sometimes associated with chronic inflammation, such as seen with rheumatic diseases. This case highlights the need for understanding TPO so that it can be differentiated from potentially serious conditions such as necrotizing granulomatous diseases, invasive infections, and cancer.

## Case

An 85-year-old Caucasian male ex-smoker with a history of chronic obstructive pulmonary disease and bronchiectasis presented to clinic with 2 to 3 weeks of gradual onset of vocal hoarseness. He had no associated symptoms and denied any worsening of his baseline dyspnea, cough or sputum production. He also denied recent trauma, upper respiratory infections, fevers, chills, sweats, anorexia, or weight loss.

In addition to chronic obstructive pulmonary disease and bronchiectasis, his past medical history was significant for aortic stenosis, coronary artery disease, dyslipidemia, hypertension, carotid stenosis, peripheral vascular disease, anemia, diabetes mellitus, and gastroesophageal reflux disease. The patient reported a prior positive PPD for which he was treated with isoniazid for 9 months without any complications. He used to smoke 1 to 3 packs per day for 37 years and quit 26 years prior to presentation.

**Figure 1.**
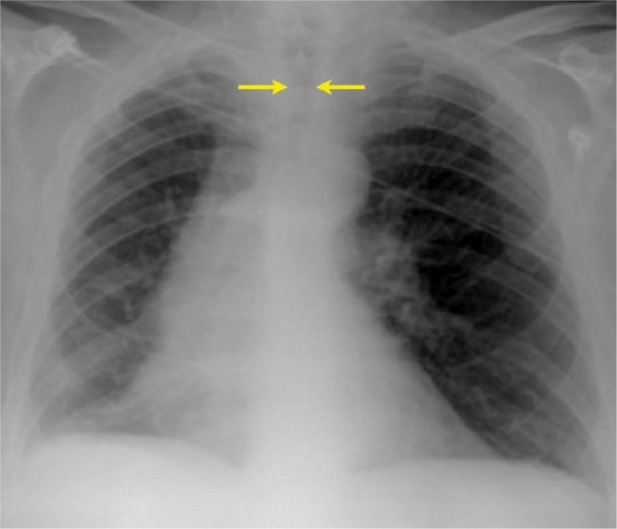
Chest radiograph is apparent for luminal narrowing of the trachea.

**Figure 2.**
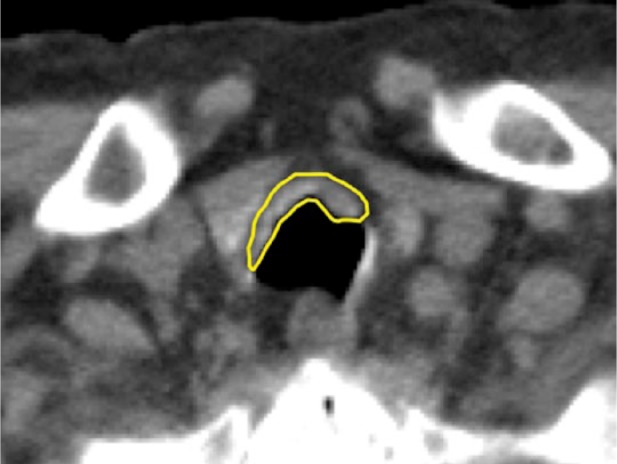
The chest CT shows a subglottic mass and anterior tracheal wall thickening with some calcifications, circumscribed by the yellow line.

**Figure 3.**
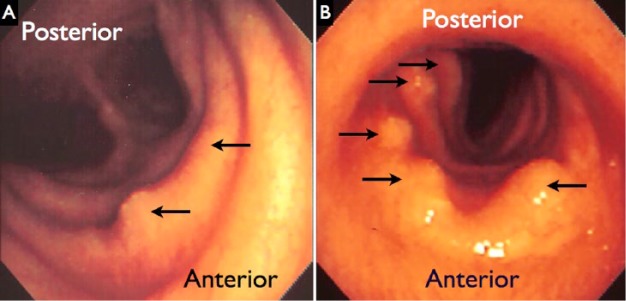
Panel A shows the proximal trachea below the true vocal cords. Panel B shows the distal trachea just above the main carina. Multiple submucosal nodules are noted by the arrows.

Physical exam did not reveal any head or neck lymphadenopathy or masses. There was no evidence of stridor. Lung exam showed scattered bilateral wheezing and rhonchi, but no crackles. Cranial nerve neurological exam was also without gross deficits. Laboratory data were unremarkable except for leukocytosis (16 100/µL) and chronic renal insufficiency (blood urea nitrogen 59 mg/dL, creatinine 3.3 mg/dL). Chest radiograph and computed tomography (CT) scan of the chest are shown in Figure 1 and Figure 2, respectively. Bronchoscopy findings are shown in Figure 3.

### Diagnosis

Tracheopathia osteoplastica (TPO).

### Patient Evaluation

The chest x-ray was significant for some narrowing of the tracheal lumen in the subglottic trachea and main trachea (Figure 1). CT scan showed a subglottic mass and mucosal irregularity with calcification in anterior and lateral walls of the trachea with intact posterior membranous wall (Figure 2). Laryngoscopy performed by our otolaryngology specialist showed mild deformity of right vocal cord, but no supraglottic abnormalities or lesions (not shown).

Bronchoscopic evaluation (Figure 3) showed normal and symmetric vocal cords; the right vocal cord deformity seen earlier with laryngoscopy was not seen during bronchoscopy. Beyond the true vocal cords, multiple submucosal nodules overlying the tracheal and cricoid cartilaginous rings were seen, most prominent over the cricoid. The trachea was saber shaped. The right and left bronchial tree were normal except for mild pitting, with evidence of mild-to-moderate dynamic collapse throughout.

Figure 3 points to the submucosal nodules seen throughout the subglottic space and proximal trachea. The saber shape of the trachea is seen in Panel A, while Panel B shows a number of submucosal lesions in the distal trachea just above the main carina.

## Discussion

Tracheopathia osteoplastica (TPO) is a rare disorder, detected in approximately 1 in every 2000 to 5000 patients who undergo bronchoscopy.^1,2^ First descriptions of TPO have been attributed to works by Rokitansky in 1855, Luschka in 1856, and Wilks in 1857.^3^ It usually manifests in adults in the sixth decade of life or later, with some pediatric cases having been reported.^1,2^ Typical presenting symptoms relate to airway stenosis and are thus nonspecific and can include dyspnea, hoarseness, cough, stridor, wheezing, or dysphagia. Hemoptysis may develop if mucosal ulcerations develop or submucosal vascular beds are compromised.

The clinical course is characterized by proliferation of bony and cartilaginous spurs leading to airway stenosis. Multiple submucosal cartilaginous and osseous nodules can develop in the respiratory tract and may involve the entire trachea and mainstem bronchi. The posterior tracheal membrane or wall (posterior pars membranacea) is typically spared, and the overlying mucosa is normal in appearance. Findings from CT or bronchoscopy characteristically show airway narrowing due to these cartilaginous and bony spicules projecting into the airway lumen. The bronchoscopic findings have been described as having the appearance of cobblestone or a stalactite cave.^2^

The pathogenesis of TPO, though largely mysterious, involves cartilaginous supporting rings of airways and thus may extend from trachea to main bronchi.^4^ The disease is thought to begin with a persistent purulent tracheitis, which, probably owing to calciphylaxis, causes accumulation of calcium salts in the tracheal mucosa. Cartilage and bone later develop around these accumulations. Calcium and phosphorus metabolism is not disturbed, and no immunological aberrations were found in any of the patients as per 30 cases that were diagnosed and studied at Department of Otolaryngology of Kuopio University.^1^ TPO can be familial and is sometimes associated with chronic inflammation or trauma. Although the pathogenesis of TPO has not been elucidated, there are case reports in patients with known rheumatic disease, which raises suspicion of a rheumatologic or immune mediated etiology as one possibility.^5^

The differential diagnosis for airway thickening with stenosis includes malignancy (bronchogenic, laryngeal, esophageal and thyroid carcinomas, Hodgkin’s lymphoma); tracheobronchial amyloidosis; atrophic and relapsing polychondritis; idiopathic laryngeal tracheal stenosis; a granulomatous pathology such as tuberculosis, Wegener’s granulomatosis, or sarcoidosis; mucopolysaccharidoses; Sjogren’s syndrome; and saber sheath trachea. Some may also consider respiratory papillomatosis. The diagnosis is seldom made because of the chronic and asymptomatic nature of the condition. More than 90% of the cases are diagnosed at postmortem examination.^2^

Diagnosis is usually made with bronchoscopy, pathological examination of biopsy specimens, CT, and magnetic resonance imaging of the chest. Although in our case TPO was limited to the upper trachea, in some reports it occurs more commonly in the lower three-fourths of the trachea and can extend to involve the first portion of the major bronchi. TPO may also be diagnosed incidentally, and in one case was discovered at the time of a difficult intubation in a patient with no prior symptoms despite the presence of extensive endotracheal and bronchial TPO lesions.^6^

The disease closest to TPO with respect to pathology is perhaps tracheobronchial amyloidosis. However, the bronchoscopic appearance easily differentiates it from TPO because amyloidosis involves the posterior aspect of the trachea with grayish submucosal plaques that bleed on contact.^7^ Sparing of the posterior (membranous) wall of the trachea has not been reported in cases of tracheobronchial amyloidosis, and in this case, was an important anatomical finding that allowed us to rule out amyloidosis. Also, it is unlikely that sarcoidosis caused the tracheal stenosis in our patient. He had no evidence of involvement of the lungs, mediastinum, eyes, or skin. When sarcoidosis results in tracheal stenosis, the larynx is usually involved as well. Further analyses also did not support a diagnosis of Wegener’s granulomatosis in this case including negative p- and c-ANCA blood tests. Some may also consider a differential diagnosis of papillomatosis. However, papillomatosis is rarely isolated to the trachea, and the bronchoscopic findings here do not have the classical appearance of papillomatosis. Papillomatosis frequently presents as “grape-like” aggregates having a characteristic glossy mucosal rounded or polypoid appearance and importantly without evidence of calcification. In addition, papillomatosis is not limited to the anterior trachea or cartilaginous rings.^8^ TPO as in this case has no such features. These points highlight the importance of bronchoscopy, where indicated, in the diagnosis of TPO to differentiate it from other diseases.

In most cases, visualization of the distinct multiple cartilaginous or bony lesions sparing the posterior aspect of the airways with bronchoscopy or CT is sufficient to diagnose TPO.^9^ Histological examination of the submucosal nodules, although largely unnecessary for diagnosis, usually demonstrates normal mucosa with focal calcification and heterotopic bone formation,^2^ and benign cartilaginous or ossified lesions composed of mature, lamellar bone with hematopoietic components.^10^ Pulmonary function testing would reveal either a normal or an obstructive pattern.^11^ Abnormalities of pulmonary function tests (PFTs) are dependent on the degree of involvement of the airway lumen.^2^ Our patient had PFT done few years prior to diagnosis, which at that time showed mild airway obstruction with low diffusion capacity. After TPO was diagnosed, no further PFTs were performed.

Treatment of TPO is symptomatic ranging from conservative treatment, bronchoscopic intervention, to operative correction. Some patients have no symptoms and others present with severe dyspnea, hemoptysis, pneumonitis, or massive tracheal hemorrhage causing respiratory failure. For most cases treatment is seldom necessary. Asymptomatic patients should be treated conservatively with regular follow-up as the natural history of the disease is relatively benign with slow progression.^4^ Symptomatic treatment involves regular tracheobronchial toilet and treating infection episodes with the appropriate antibiotics. TPO usually follows a benign and slowly progressive course with some reported cases of obstructive pneumonia complicating it. In some cases of chronic cough lasting years, treatment with airway clearance devices and a nasal corticosteroid spray resulted in the resolution of cough.^12^ Conversely, in severe cases, bronchoscopic removal of obstructing excrescences and surgery has been performed with therapeutic effect.^13^

Surgical intervention is indicated to treat symptoms of severe airway stenosis; for short segmental stenosis, bronchoscopic curettage and resection of tracheal nodules have been reported as an effective treatment in relieving airway obstruction.^1,2^ In patients experiencing frequent infections and/or severe airway obstruction, cryotherapy, excision with Nd:YAG laser or external radiation may be used, or the obstructing lesion may be removed via bronchoscopy.^2^ Tracheal and bronchial stenting via flexible bronchoscopy has also been reported to successfully treat TPO.^14^ Grillo and Wright reported 4 cases of TPO treated effectively with linear tracheoplasty.^14^ Stent removal after firm healing produced long-term correction of stenosis in 3 of 4 patients, examined up to 12 years.^14^

In extreme and rare cases, rapid progression has led to severe symptoms and even death.^15^ When respiratory dysfunction occurs, treatment options to relieve airway stenosis include rigid bronchoscopy with dilation and removal of the obstructing spurs, or complete segmental tracheal resection.^15^ Nevertheless, the experience in treating this rare condition is still limited in literature. Our patient was offered no treatment. He died a few months later from unrelated causes.

In summary, TPO is a rare disease of unclear etiology. Symptoms may range from a completely silent condition to life-threatening respiratory failure. However, most often TPO has a benign course. Diagnosis is made based on radiological and bronchoscopic findings. Biopsy and histopathology is desirable but not necessary for diagnosis. Management is conservative and focused on alleviating symptoms. Successful surgical intervention for airway obstruction has been described and continues to be experimental and on a case-by-case basis. This case underpins that a symptom as common and nonspecific as vocal hoarseness can lead to interesting diagnoses like TPO. Understanding TPO also allows it to be differentiated from potentially serious conditions such as necrotizing granulomatous diseases, invasive infections, and cancer.
